# Characteristics of electroencephalographic changes after hemispheric disconnection surgery in patients with drug-resistant epilepsy

**DOI:** 10.1186/s42494-026-00269-z

**Published:** 2026-08-01

**Authors:** Xinghua Cui, Zaifen Gao, Shuhua Chen, Lu Liu, Jianguo Shi, Guifu Geng, Xiuli Zhan

**Affiliations:** 1 Epilepsy Center, Jinan Children’s Hospital (Children’s Hospital Affiliated to Shandong University), Jinan, 250022 China; 2 Department of Neurology, Beijing Children’s Hospital, Capital Medical University, Beijing, 100045 China

**Keywords:** Hemispheric disconnection surgery, Drug-resistant epilepsy, Video-EEG, Burst-suppression-like pattern, Prognosis

## Abstract

**Background:**

Hemispheric disconnection surgery is a safe and effective therapeutic modality for drug-resistant epilepsy (DRE) secondary to hemispheric or multilobar lesions, while the dynamic evolutionary characteristics of postoperative electroencephalogram (EEG) and its clinical prognostic value remain unclear with no relevant systematic follow-up reports currently available. This study aims to retrospectively analyze EEG changes following hemispheric disconnection surgery in patients with DRE and to assess their clinical significance.

**Methods:**

We enrolled patients with DRE who underwent hemispheric disconnection surgery. EEG data were collected preoperatively and at 3 months, 1 year, and 2 years postoperatively. We analyzed EEG characteristics (including background activity and interictal and ictal patterns) at each time point and evaluated their correlation with prognosis.

**Results:**

Sixteen patients met the inclusion criteria; 14 (87.5%) had no seizure recurrence within the 2-year follow-up period. EEG analysis at postoperative time points showed that: (1) The EEG background activity on the ipsilateral side was dominated by slow waves and low-voltage activity. The proportion of low-voltage background activity gradually increased within 1 year, and slow waves became the predominant pattern thereafter. While, the contralateral background activity normalized within 1 year. (2) Epileptiform discharges on the ipsilateral side initially increased then decreased, accompanied by a reversible burst-suppression-like pattern. Epileptiform discharges on the contralateral side gradually decreased and disappeared in most patients (8/11, 72.7%). (3) In this study, seizure recurrence occurred in 2 patients, which was associated with incomplete disconnection and complications. Seizures were subsequently controlled after reoperation. (4) No postoperative EEG features were significantly correlated with prognosis (all *P* > 0.05).

**Conclusions:**

Following hemispheric disconnection, dynamic alterations are observed in bilateral EEG recordings. A postoperative burst-suppression-like pattern is commonly observed on the ipsilateral side. In this small-sample study, no significant association was found between postoperative EEG characteristics and prognosis.

**Supplementary Information:**

The online version contains supplementary material available at 10.1186/s42494-026-00269-z.

## Background

Hemispheric lesions are a common etiology of drug-resistant epilepsy (DRE) in children [1, 2]. Hemispheric disconnection surgery is a safe and effective treatment for DRE associated with hemispheric or multilobar lesions [3, 4], accounting for approximately 20–40% of epilepsy surgeries [5]. Early surgical intervention is associated with improved cognitive and motor outcomes in pediatric patients [4, 6]. Video-electroencephalogram (VEEG) is an important tool for preoperative evaluation and postoperative assessment of surgical efficacy and prognosis in patients with epilepsy [7]. However, there have been few reports on the dynamic follow-up of electroencephalogram (EEG) after hemispheric disconnection surgery. This study retrospectively analyzed the clinical and EEG data of 16 patients who underwent hemispheric disconnection surgery at the Epilepsy Center of our hospital, aiming to summarize the dynamic evolution of postoperative EEG and explore its clinical significance.

## Subjects and methods

### Study design

The present study was a retrospective, single-center descriptive study. It was approved by the Hospital Ethics Committee (SDFE-IRB/T-2025115) and conducted in strict accordance with the Declaration of Helsinki. Given the retrospective nature of the study, informed consent was not required, as it was waived by the Ethics Committee of the Jinan Children’s Hospital approval regulations.

### Study subjects

Patients with DRE who underwent hemispheric disconnection surgery at the Epilepsy Center of Jinan Children’s Hospital between November 2020 and May 2023 were enrolled.

#### Inclusion criteria

1. Patients met the 2010 International League Against Epilepsy (ILAE) diagnostic criteria for DRE [8].
2. Multidisciplinary preoperative evaluation indicated that the epileptogenic zone was consistent with the lesion in the affected hemisphere, and all patients underwent hemispheric disconnection surgery.
3. The postoperative follow-up duration was ≥ 2 years.

#### Exclusion criteria

1. Patients had other potential etiologies for DRE, such as metabolic disorders, mitochondrial encephalopathy, nephropathy, hepatopathy, or vasculitis.
2. Patients were lost to follow-up.

### Methods

#### Preoperative evaluation

All patients underwent a detailed multidisciplinary preoperative evaluation for epilepsy. Clinical data included age, sex, duration of epilepsy, age at surgery, positive neurological signs, etiology, assessment of developmental and motor function, cranial imaging findings, VEEG results, and intraoperative pathological findings (where available). All patients underwent preoperative 3.0 T magnetic resonance imaging (MRI) with the Harmonized Neuroimaging of Epilepsy Structural Sequences (HARNESS) protocol and fluorodeoxyglucose positron emission tomography (FDG-PET); imaging findings revealed unilateral hemispheric structural abnormalities in all patients. The treatment plan was formulated through multidisciplinary discussion among pediatric epilepsy specialists, epilepsy surgeons, and neurophysiologists.

#### Surgical method

All patients underwent hemispheric disconnection surgery performed by experienced epilepsy surgeons. A brief description of the surgical procedure is as follows: Under the operating microscope, the opercular tissue was resected, and the basal frontal region was disconnected. The lateral ventricle was then accessed, followed by the transection of the corpus callosum. The connections between the fronto-parieto-occipital lobes and the basal ganglia were disconnected, as were the connections involving the insular cortex and the temporal lobe. The posterior hippocampus and amygdala were individually disconnected and resected along the periphery of the basal ganglia.

#### VEEG monitoring and interpretation

VEEG monitoring was performed using a 32-channel digital multifunctional electroencephalography system (Nihon Kohden, Japan). Scalp disk electrodes were placed in strict accordance with the International 10–20 System, with synchronous recording of electrocardiography (ECG) and electromyography (EMG). The VEEG acquisition parameters were set as follows: low-frequency filter (LF) 0.5 Hz, high-frequency filter (HF) 70 Hz, notch filter 50 Hz, and sensitivity 10 µV/mm. Monitoring duration was ≥ 24 h preoperatively and ≥ 3 h at each postoperative time point. All VEEG recordings included at least one full sleep–wake cycle when feasible, with simultaneous video monitoring for clinical seizure correlation. The quantification of interictal epileptiform discharges was performed during both wakefulness and sleep, with discharge burden expressed as the number of discharges per hour to standardize comparisons across recordings of different durations. VEEG examinations were conducted at predefined time points: preoperatively, followed by 3 months, 1 year, and 2 years post-surgery. VEEG analysis included evaluation of background activity as well as interictal and ictal patterns. All recordings were independently reviewed by two experienced neurophysiologists at the attending physician level or above, with findings documented separately. Inter-rater reliability was assessed using Cohen’s kappa coefficient. Any discrepancies were resolved through departmental discussion, and a consensus interpretation was adopted.

#### Postoperative MRI assessment

Postoperative MRI scans were performed at 3–6 months after surgery in all patients to evaluate surgical outcomes and detect complications such as hydrocephalus or residual connections. The imaging protocol included T1-weighted imaging (T1WI), T2-weighted imaging (T2WI), and fluid-attenuated inversion recovery (FLAIR) sequences. Qualitative assessment of disconnection completeness was performed by experienced epilepsy surgeons based on visualization of the surgical disconnection planes. However, owing to clinical practice constraints, systematic quantitative volumetric analysis of residual tissue or standardized grading of disconnection completeness was not uniformly implemented across all patients.

#### Follow-up and prognosis evaluation

All patients were followed up for at least 2 years via telephone and/or outpatient visits. Postoperative assessments included seizure status, neurological function, VEEG, and cranial MRI.

Seizure prognosis was evaluated using the Engel classification: Class I (seizure-free), Class II (very rare seizures: 1–2 per year), Class III (≥ 75% reduction in seizure frequency), and Class IV (no improvement). Engel Class I was defined as seizure-free, while Classes II–IV were defined as seizure recurrence. Neurological function was assessed at the last follow-up using three indicators [5]: (1) independent ambulation (yes/no); (2) voluntary grasp of the hemiparetic hand (yes/no); (3) language ability (ability to speak short sentences or age-appropriate utterances), assessed only in patients aged ≥ 2 years.

#### Statistical methods

Statistical analyses were performed using SPSS 26.0 software (IBM Corp., Armonk, NY, USA). Measurement data were expressed as mean ± standard deviation and median (range), while categorical data were presented as frequencies and percentages. Owing to the limited sample size, Fisher’s exact test was used to assess associations between categorical variables, and the phi coefficient was calculated to evaluate the strength of these associations. Given the exploratory, hypothesis-generating nature of this study and the limited number of comparisons, *P*-values are presented uncorrected; however, findings should be interpreted with caution due to the potential for type I error inflation. To contextualize the negative findings in this small cohort, a post-hoc power analysis was conducted using G*Power 3.1 software [9]. Parameters for the exact test included a total sample size of *n* = 16, an event rate of 12.5% (2 recurrence events), a target odds ratio of 3.0 (representing a clinically meaningful effect), and *α* = 0.05 (two-tailed). Inter-rater reliability for EEG classification was assessed using Cohen’s kappa coefficient, interpreted according to standard criteria [10]. Pre- and postoperative functional outcomes (speech, ambulation, and grasp) were compared using the McNemar test. Statistical significance was defined as *P* < 0.05.

## Results

### Clinical characteristics

A total of 16 patients were enrolled, with a male-to-female ratio of 3:5. Handedness distribution was left-handed: right-handed: undetermined = 4:7:5. The mean age at surgery was 45.9 ± 22.3 months, and the mean duration of epilepsy was 22.1 ± 19.5 months. Seizure types were classified according to the 2025 ILAE criteria [11] as follows: focal seizures in 13/16 (81.3%) patients, generalized seizures in 2/16 (12.5%), and focal plus generalized seizures in 1/16 (6.3%). All patients had structural epileptogenic etiologies, including developmental etiologies in 11/16 (68.8%), acquired etiologies in 4/16 (25.0%), and combined developmental and progressive etiologies in 1/16 (6.3%). FDG-PET scans in all patients showed decreased metabolism in the disconnected hemisphere (Table 1 and Supplementary Table 1).

**Table 1:** Clinical characteristics of the patients

Variables	Statistical value
**Total (** ***n*** **)**	16
**Sex (No. of patients**, ***%*)**	
Male	6 (37.5*%*)
Female	10 (62.5*%*)
**Age at surgery (months)**	
* Mean ± SD*	45.9 ± 22.3
* Median (min–max)*	47.50 (4–77)
**Duration of epilepsy (months)**	
* Mean ± SD*	22.1 ± 19.5
* Median (min–max)*	13 (2–60)
**Seizure type (No. of patients**, ***%*)**	
Focal (including electrographic seizures)	13/16 (81.3*%*)
Generalized	2/16 (12.5*%*)
Focal + generalized	1/16 (6.3*%*)
**MRI lesion location (No. of patients**, ***%*)**	
Left	7/16 (43.8*%*)
Right	9/16 (56.3*%*)
**Seizure etiology (No. of patients**, ***%*)**	
Developmental etiologies	11/16 (68.8*%*)
Acquired etiologies	4/16 (25.0*%*)
Developmental and progressive etiologies	1/16 (6.3*%*)

### Postoperative seizure control and functional recovery

All patients underwent hemispheric disconnection surgery, involving the right hemisphere in 9 cases (56.3 %) and the left hemisphere in 7 cases (43.8%). The mean postoperative follow-up duration was 41.1 ± 7.7 months. Postoperatively, patients were routinely maintained on 1–2 anti-seizure medications (ASMs). For patients who remained seizure-free for at least 1 year postoperatively, with no significant increase in epileptiform discharges in the contralateral hemisphere, gradual tapering and discontinuation of ASMs were undertaken based on clinical evaluation and the preferences of patients and their families. In this cohort, 1 patient experienced seizure recurrence at 3 months and 1 at 1 year after surgery; 15 patients (93.8%) achieved Engel Class I at both time points. At the last follow-up, all 16 patients (100.0%) were classified as Engel Class I. Fourteen patients (87.5%) were undergoing ASM tapering, 11 (68.8%) could walk independently, 6 (37.5%) had voluntary grasp function, and 6 (37.5%) could speak short sentences or had age-appropriate language function. However, no statistically significant differences were found compared with preoperative values (all *P* > 0.05) (Table 2).

**Table 2:** Surgical characteristics and outcomes of the patients

Variables	Statistical value
**The operative side (No. of patients, %)**	
Left	7/16 (43.8*%*)
Right	9/16 (56.3*%*)
**Follow-up duration (months)**	
* Mean± SD*	41.1 ± 7.7
* Median (min–max)*	42 (27–55)
**Engel Class (No. of patients**, ***%*)**	
Class I	16/16 (100.0*%*)
Class II	0/16 (0.0*%*)
Class III	0/16 (0.0*%*)
**Postoperative functional recovery** **(No. of patients**, ***%*)**	
Independent ambulation	11/16 (68.8*%*)^a^
Voluntary hand movement	6/16 (37.5*%*)^a^
Speech	6/16 (37.5*%*)^a^

Note: a indicates *P* > 0.05 compared with preoperative values

### Inter-rater reliability

A total of 64 EEG recordings (16 patients × 4 time points) were independently classified by two neurophysiologists. Cohen’s kappa coefficients demonstrated excellent inter-rater agreement across all EEG classification categories. For background activity classification, *κ* = 0.887 (95% CI: 0.782–0.992, *P* < 0.001), indicating almost perfect agreement. For the presence of interictal epileptiform discharges, *κ* = 0.889 (95% CI: 0.767–1.000, *P* < 0.001), also indicating almost perfect agreement. For interictal epileptiform discharge distribution patterns, *κ* = 1.000 (95% CI: 1.000–1.000, *P* < 0.001), indicating perfect agreement. For seizure onset/ictal pattern classification, *κ* = 1.000 (95% CI: 1.000–1.000, *P* *<* 0.001), also indicating perfect agreement. For burst-suppression-like pattern identification, *κ* = 0.868 (95% CI: 0.724–1.000, *P* < 0.001), indicating almost perfect agreement. These results demonstrate the high reliability of EEG interpretation between the two independent reviewers.

### Preoperative VEEG

Background activity: Twelve patients (75.0%) demonstrated continuous low- to moderate-amplitude (< 150 µV) theta (4–7.5 Hz) and delta (0.3–3.5 Hz) activity (hereafter collectively referred to as slow waves) in the ipsilateral hemisphere. Two patients (12.5%) showed continuous theta and delta activity with amplitudes < 20 µV (hereafter referred to as low-voltage activity) in the ipsilateral hemisphere. The background activity was normal in 2 patients (12.5%). On the contralateral side, only 3 patients (18.8%) presented with slow waves, while background activity was normal in the remaining cases.

Interictal activity: Epileptiform discharges were recorded in the ipsilateral hemisphere in all patients. Regional epileptiform discharges in the contralateral hemisphere were detected in 10 patients (62.5%), while generalized discharges were observed in 3 patients (18.8%).

Ictal activity: Ictal seizures were recorded preoperatively in all 16 patients (100.0%). Twelve patients (75.0%) had focal seizures originating from the ipsilateral hemisphere, 2 patients (12.5%) had generalized seizures, 1 patient (6.3%) exhibited both generalized seizures and focal seizures originating from the ipsilateral hemisphere, and 1 patient (6.3%) showed only electrographic seizures arising from the ipsilateral hemisphere (Table 3, Supplementary Table 2).

**Table 3:** Summary of preoperative VEEG characteristics

Category	Ipsilateral side‌	‌Contralateral side ‌
**Background**	Slow waves: 12 cases (75.0%)	Slow waves: 3 cases (18.8%)
	Low-voltage activities: 2 cases (12.5%)	Normal: 13 cases (81.3%)
	Normal: 2 cases (12.5%)	-
**Interictal**	Regional discharges: 7 cases (43.8%)	Regional discharges: 8 cases (50.0%)
	Multifocal discharges: 9 cases (56.3%)	Generalized discharges*: 1 case (6.3%)
	-	Regional+ generalized discharges*: 2 cases (12.5%)
	-	Normal: 5 cases (31.3%)
**Seizure onset**	Ipsilateral focal-onset: 12 cases (75.0%)	-
	Generalized onset: 2 cases (12.5%)	-
	Generalized + Ipsilateral focal-onset: 1 case (6.3%)	-
	Ipsilateral focal-onset electrographic seizures: 1 case (6.3%)	-

Note: Background slow waves refer to low- to moderate-amplitude theta and delta waves; background low-voltage activities refer to theta and delta waves with amplitudes < 20 µV; * indicates generalized discharges involving both hemispheres; “–” indicates no relevant data

### Postoperative VEEG

Four patients underwent follow-up VEEG 1 month after surgery. Among them, ipsilateral epileptiform discharges were reduced compared with preoperative levels in 3 patients. One patient showed a marked increase in discharges and exhibited a burst-suppression-like pattern: the burst phase consisted of medium- to very high-amplitude slow waves mixed with spikes and sharp waves, lasting 0.5–2 s, while the suppression phase showed amplitudes < 20 µV and lasted 1–3 s (hereafter collectively referred to as the burst-suppression-like pattern).

Follow-up VEEG at 3–4 months postoperatively: (a) Background activity in the ipsilateral hemisphere consisted of either persistent slow waves (8 cases, 50.0%) or low-voltage activity (8 cases, 50.0%) in all patients, while slow waves in the contralateral hemisphere were reduced compared with preoperative findings. (b) Increased epileptiform discharges with higher amplitude in the ipsilateral hemisphere were observed in 11 patients (68.8%), and 9 patients (56.3%) exhibited a burst-suppression-like pattern. Compared with preoperative findings, epileptiform discharges decreased in 4 patients (25.0%) and disappeared in 1 patient (6.3%). In the contralateral hemisphere, among the 11 patients with preoperative epileptiform discharges, the EEG demonstrated a reduction in discharge burden in 8 patients (72.7%) and complete resolution in the remaining 3 (27.2%). (c) Case 14 developed hydrocephalus and recurrent epilepsy postoperatively, and seizures were well controlled after lateral ventriculoperitoneal shunt (LVPS) surgery.

Follow-up VEEG at 1 year postoperatively: (a) The proportion of low-voltage activity in the ipsilateral hemisphere increased (11/16, 68.8%), while the proportion of slow waves decreased (5/16, 31.3%). All 3 patients with abnormal contralateral background activity had returned to normal. (b) Compared with findings at 3–4 months postoperatively, ipsilateral epileptiform discharges increased in 7 patients (43.8%), and the burst-suppression-like pattern became more prominent or newly emerged in 6 patients (37.5%). Contralateral epileptiform discharges increased in 3 patients, while complete resolution was observed in the remaining patients. (c) Electrographic seizures in the ipsilateral hemisphere were observed in Case 1. In Case 15, postoperative seizure recurrence occurred 1 year after surgery. Postoperative MRI revealed residual white matter fiber tracts in the surgical area, while EEG demonstrated persistent abundant epileptiform discharges without a burst-suppression-like pattern. Based on combined clinical, EEG, and MRI evidence, incomplete disconnection was inferred. The patient subsequently underwent repeat disconnection surgery, after which seizures were controlled. This case highlights the clinical utility of integrating EEG findings with neuroimaging to identify incomplete disconnection, while also emphasizing the need for systematic imaging assessment to strengthen such inferences.

**Fig. 1:**
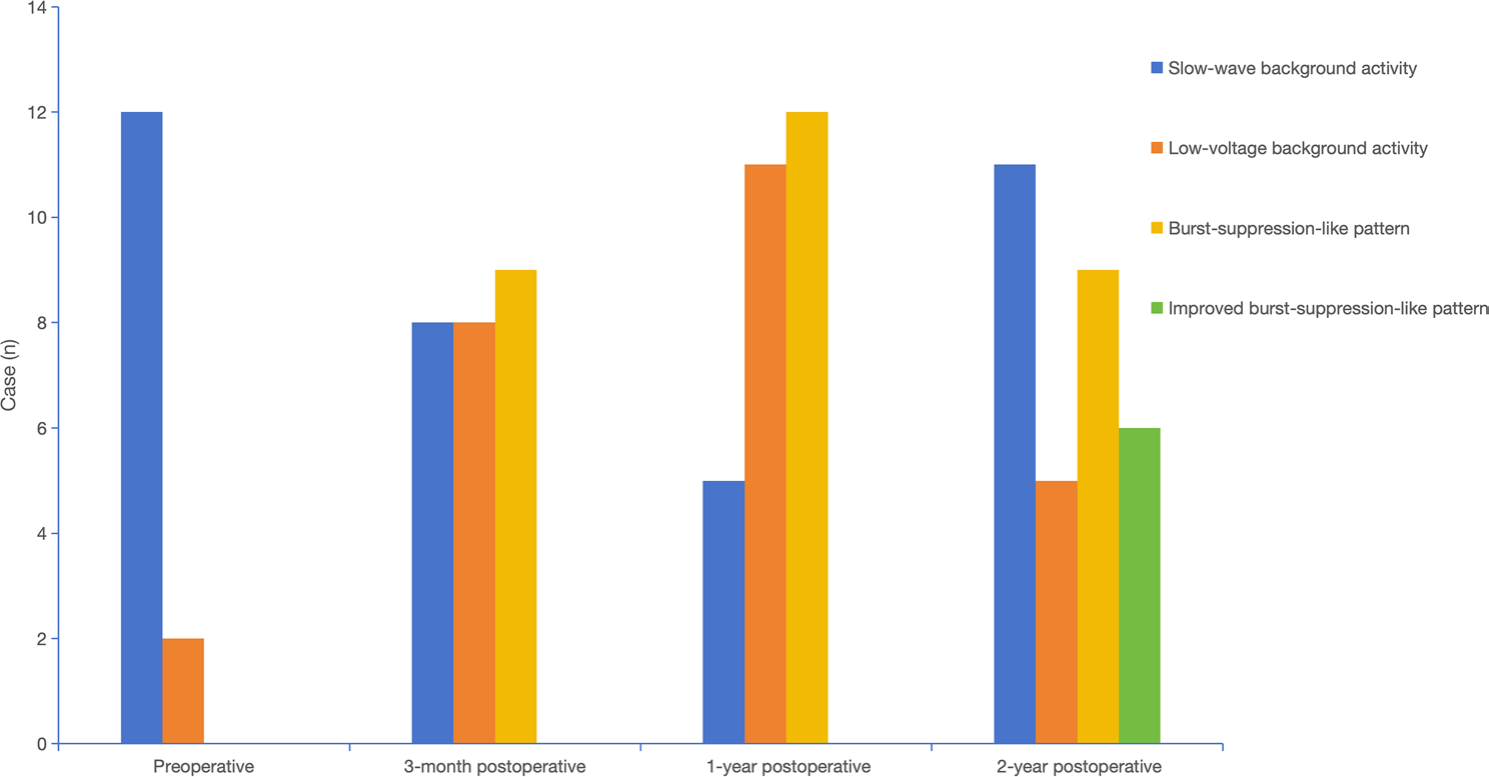
Summary of EEG features over follow-up on the ipsilateral hemisphere preoperatively and postoperatively

**Fig. 2:**
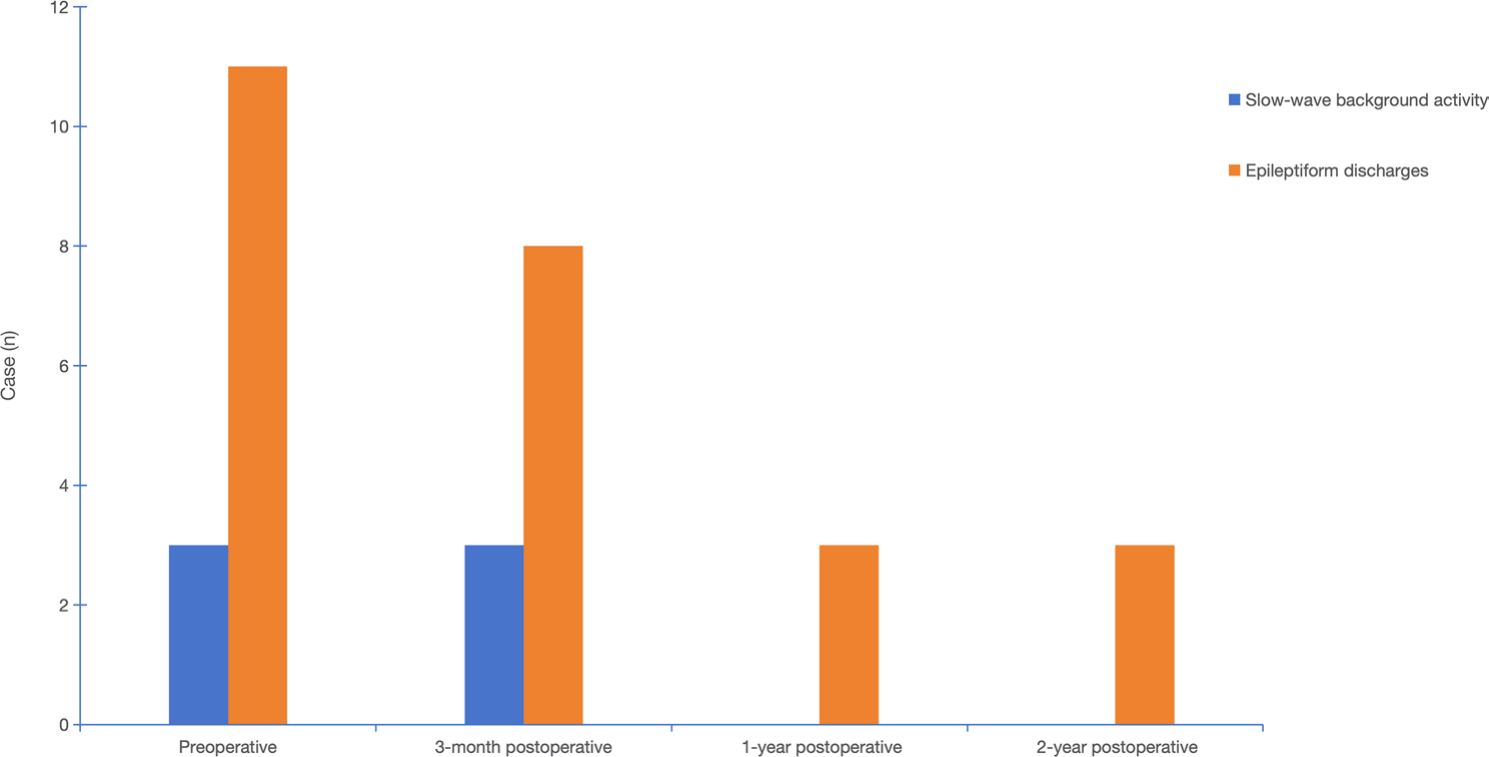
Summary of EEG features over follow-up on the contralateral hemisphere preoperatively and postoperatively

Follow-up VEEG at 2 years postoperatively: (a) The overall amplitude of background activity in the ipsilateral hemisphere was higher than before. Predominantly slow-wave activity was observed in 11 patients (68.8%), while predominantly low-voltage activity was observed in 5 patients (31.3%). (b) The number and amplitude of epileptiform discharges in the ipsilateral hemisphere decreased, with waveforms and discharge burden tending to stabilize, and the burst-suppression-like pattern gradually reduced. Among the 16 patients, the burst-suppression-like pattern showed improvement in 6 (37.5%), characterized by ≥ 30% reduction in burst-suppression-like pattern amplitude ratio or ≥ 50% decrease in overall burst-suppression-like pattern burden. Additionally, the pattern completely resolved in 3 patients (18.8%). Among the 3 patients with contralateral epileptiform discharges, 2 showed a decrease in discharge frequency, while 1 showed no significant change. (c) At the 2-year follow-up, all patients were seizure-free (Figs. 1 and 2, and Supplementary Table 3).

Among the 16 patients in this study, the co-occurrence rate of low-voltage activity and burst-suppression-like patterns in the ipsilateral hemisphere postoperatively was 56.3% (9/16). Fisher’s exact test showed no statistically significant difference in the incidence of burst-suppression-like patterns according to whether the postoperative ipsilateral background was dominated by low-voltage activity (*P* > 0.05, *φ* = −0.277). No statistically significant differences in postoperative seizure recurrence were observed with respect to whether the background was dominated by low-voltage activity (co-occurrence rate: 68.8%, *P* > 0.05, *φ* = −0.182), the presence or absence of a burst-suppression-like pattern (co-occurrence rate: 68.8%, *P* > 0.05, *φ* = 0.218), or the presence or absence of preoperative contralateral epileptiform discharges (co-occurrence rate: 56.3%, *P* > 0.05, *φ* = −0.255) (Table 4). These findings suggest that the above EEG characteristics are not associated with prognosis. However, the limited sample size resulted in low statistical power for these analyses. Therefore, these null findings should be interpreted with caution, as the study cannot distinguish between a true absence of association and a Type II error due to insufficient power.

**Table 4:** Association analysis between EEG characteristics and prognosis

Measure	Postoperative low-voltage activities		Postoperative burst-suppression-like pattern		Preoperative contralateral epileptiform discharges		
	Present/Absent		Present/Absent		Present/Absent		
Seizure-free (*n*)	11/3		11/3		9/5		
Recurrence (*n*)	2/0		1/1		2/0		
*P* value	1.000		0.450		1.000		0.529^a^
*φ* value	−0.182		0.218		−0.255		−0.277^b^

Notes: *P* < 0.05 indicates a statistically significant difference; the larger the absolute value of *φ*, the stronger the association, and the positive/negative sign indicates the direction of the association; ᵃ represents the *P* value for the association between postoperative low-voltage activity and burst-suppression-like pattern; ᵇ represents the *φ* value indicating the strength of association between postoperative low-voltage activity and burst-suppression-like pattern

### Post-hoc power analysis results

Post-hoc power analysis demonstrated that the study was severely underpowered to detect clinically meaningful associations. With a total sample size of *n* = 16 and only 2 recurrence events (12.5% event rate), the statistical power to detect a clinically meaningful association (OR ≥ 3.0) was approximately 17.2%, with a Type II error probability (*β)* of 82.8% at an *α* level of 0.05.

Post-hoc power analysis based on the effect sizes observed in Table 4 indicated that the recurrence-related analyses were statistically underpowered. The estimated statistical power was 11.2% for postoperative low-voltage activity, 14.1% for postoperative burst-suppression-like patterns, and 17.5% for preoperative contralateral epileptiform discharges, corresponding to Type II error probabilities of 88.8%, 85.9%, and 82.5%, respectively. These low power values indicate that the absence of statistically significant associations should be interpreted as inconclusive rather than as definitive evidence of no association.

### Postoperative MRI findings

Postoperative MRI was performed in all 16 patients at 3–6 months after surgery. Imaging findings demonstrated successful hemispheric disconnection in 14 patients. The ipsilateral hemisphere showed volume loss, disorganized architecture, focal parenchymal defects, and poor structural delineation. In Case 14, postoperative MRI revealed hydrocephalus requiring LVPS. In Case 15, postoperative MRI findings, together with clinical seizure recurrence and persistent ipsilateral epileptiform discharges, suggested incomplete disconnection, which was confirmed by clinical improvement following repeat disconnection surgery. The lack of standardized quantitative assessment of disconnection completeness limits the ability to correlate anatomical disconnection status with EEG burst-suppression-like patterns across the cohort.

## Typical case

**Fig. 3:**
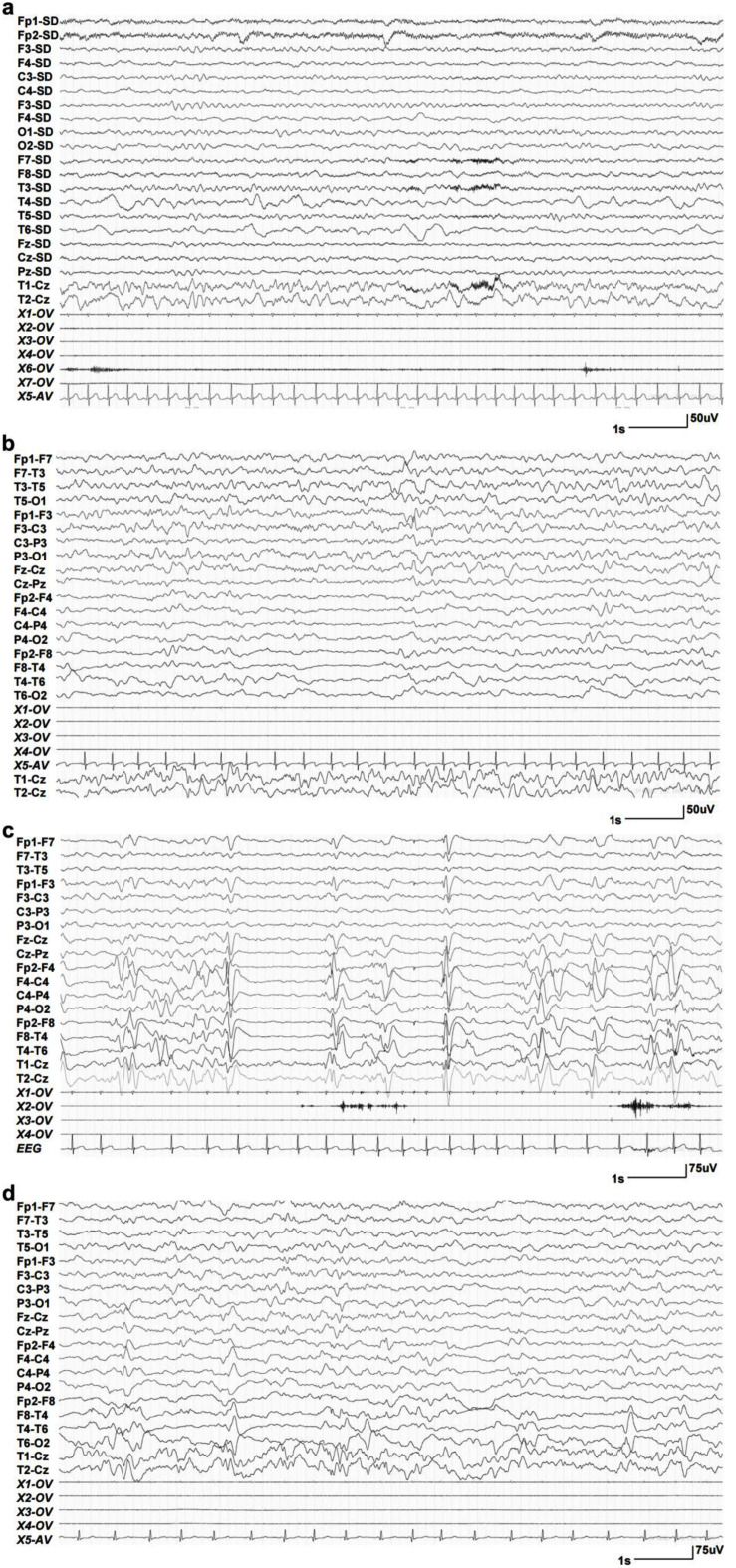
**a** Preoperative EEG showed slow waves over the right hemisphere, with intermittent medium-amplitude sharp waves and irregular slow waves during the interictal period. **b** At 4 months after surgery, the EEG revealed low-voltage background activity over the right hemisphere with occasional epileptiform discharges. **c** At 1 year after surgery, the right hemisphere EEG background showed low-voltage activity; during the interictal period, frequent high-amplitude discharges organized in a burst-suppression-like pattern were observed. **d** At 2 years after surgery, the EEG showed increased background amplitude, reduced discharges, and resolution of the burst-suppression-like pattern

**Fig. 4:**
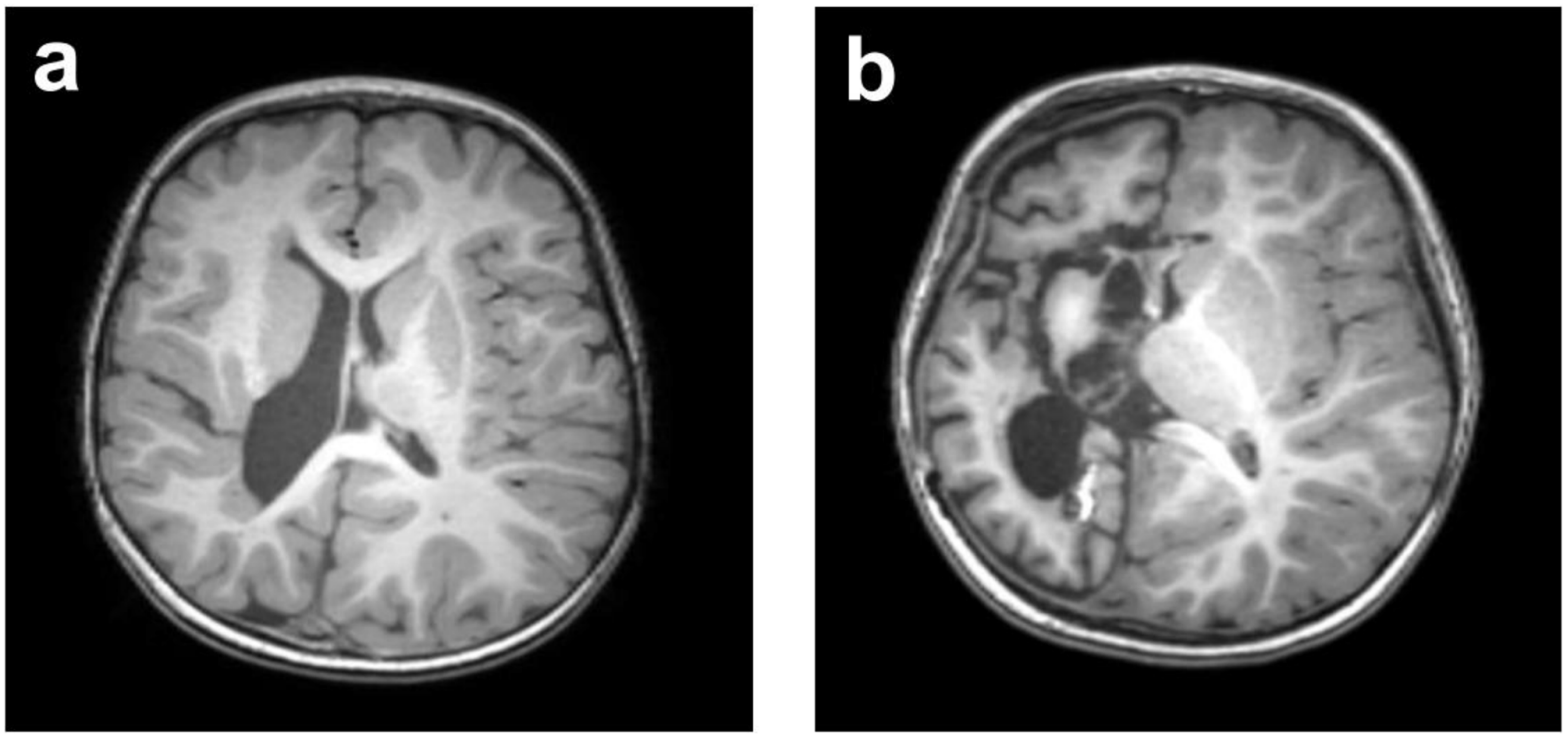
**a** Preoperative T1WI shows schizencephaly and gray matter heterotopia along the ventricular wall. **b** Postoperative T1WI shows volume loss of the right hemisphere, with disorganized architecture, focal parenchymal defects, and poor structural delineation. The right lateral ventricle is dilated with abnormal morphology. The corpus callosum is hypoplastic, and there is poor continuity of focal soft tissue in the right frontoparietal region

Case 1, diagnosed with DRE, underwent right hemispheric disconnection surgery and remained seizure-free postoperatively. Preoperative VEEG (a) demonstrated persistent slow-wave background activity over the right hemisphere, with interictal discharges consisting of sharp waves and sharp slow waves in the same region. Postoperative VEEG follow-up: at 4 months after surgery, VEEG (b) showed predominantly low-voltage background activity over the right hemisphere, with occasional epileptiform discharges; at 1 year after surgery (c), the low-voltage background persisted with frequent high-amplitude epileptiform discharges organized in a burst-suppression-like pattern; at 2 years after surgery (d), background activity had improved, with amplitudes > 20µV, epileptiform discharges were reduced, and the burst-suppression-like pattern was no longer present. Preoperative cranial MRI (a) demonstrated schizencephaly and gray matter heterotopia. Postoperative MRI (b) showed volume loss of the right cerebral hemisphere, with disorganized architecture, focal parenchymal defects, and poor structural delineation. The right lateral ventricle was dilated with abnormal morphology. The corpus callosum was hypoplastic, and there was poor continuity of focal soft tissue in the right frontoparietal region (Figs. 3 and 4).

## Discussion

Hemispheric disconnection surgery achieves seizure freedom while preserving the function of the contralateral hemisphere by interrupting neural pathways within the affected hemisphere, severing connections between the epileptogenic network and normal networks, and blocking the propagation of seizure-related abnormal discharges [12]. The seizure-free rate following this procedure has been reported to range from 66 to 90% [5, 13, 14]. In the present cohort, the seizure-free rate after hemispheric disconnection surgery was 87.5%, which is consistent with previous reports and further supports the efficacy of hemispheric disconnection surgery in the treatment of DRE.

The prognosis following hemispheric disconnection surgery is influenced by multiple factors, including age at surgery, etiology of epilepsy, presence of structural lesions or functional impairment in the contralateral hemisphere, and surgical laterality [5, 15, 16]. The high postoperative seizure-free rate observed in this study may be attributed to two main factors. First, the mean age at surgery in the present cohort was 45.9 ± 22.3 months (range: 4 months to 6 years and 5 months), reflecting a relatively young population. Previous studies have shown that pediatric patients tend to achieve better clinical outcomes [17], which may be attributed to the greater neuroplasticity of the developing brain [5]. Second, both MRI and FDG-PET findings in all patients indicated that lesions were confined to a single hemisphere. Moreover, interictal discharges in the contralateral hemisphere gradually decreased or disappeared in most patients postoperatively, suggesting that the contralateral hemisphere was not significantly affected, which may provide an important basis for a favorable prognosis. Among all enrolled patients, 7 underwent left-sided surgery and 9 underwent right-sided surgery. The postoperative seizure recurrence rate was 14.3% (1/7) in the left-sided group versus 11.1% (1/9) in the right-sided group, with a slightly higher recurrence rate observed in patients who underwent left-sided procedures. Limited by the relatively small sample size of the present cohort, the current data fail to establish a significant correlation between surgical laterality and postoperative recurrence risk. Nevertheless, the recurrence pattern observed in our study is consistent with prior research. Previous studies [15] have reported significantly higher postoperative recurrence following left-sided surgery, with statistically meaningful intergroup differences.

Given the small sample size, no stratified statistical analysis was performed for etiological subgroups in the present study, and only descriptive comparative analysis was conducted to provide preliminary evidence for future large-scale clinical investigations. Among the 16 enrolled patients, developmental etiologies accounted for the majority (11/16, 68.8%), followed by acquired etiologies (4/16, 25.0%), while 1 patient (6.3%) presented with combined developmental and progressive pathological changes. Notably, both patients who experienced postoperative seizure recurrence in this cohort had underlying developmental etiologies, with recurrence attributed to postoperative hydrocephalus and incomplete cerebral disconnection, respectively. Both patients achieved favorable seizure control after secondary reoperation. These findings suggest that patients with developmental epilepsy may be at a higher risk of postoperative recurrence. The underlying mechanisms may be explained by two key factors. First, developmental lesions often involve diffuse or multifocal cerebral abnormalities, which can limit precise delineation of surgical disconnection boundaries and increase the difficulty of achieving complete disconnection [18]. Second, this patient population may be more susceptible to postoperative complications such as hydrocephalus, which can further compromise surgical outcomes. In contrast, no postoperative recurrence was observed in patients with acquired etiologies, suggesting a more favorable overall prognosis in this subgroup. Acquired epileptogenic lesions, including perinatal brain injury and cerebral hemorrhage, are typically focal and well-circumscribed. Such localized pathological features facilitate complete surgical disconnection, reduce the risk of postoperative complications, and ultimately contribute to better seizure outcomes. Consistent with our results, previous studies have reported seizure control rates of up to 78% following hemispheric disconnection surgery for acquired epilepsy, whereas long-term seizure control rates in developmental epilepsy are relatively lower, ranging from 60% to 70% [19]. The relatively high recurrence rate of 18.2% (2/11) in the developmental subgroup in our cohort may be attributable to the early surgical experience of our center and potential case selection bias. Of note, all comparative analyses in this study remain descriptive rather than statistically conclusive. The single-center retrospective design, small sample size, and intergroup imbalance limit the generalizability of our findings. Further multicenter, large-sample prospective studies are required to validate the independent prognostic impact of different etiological categories on surgical outcomes following hemispheric disconnection surgery. Despite the limited statistical power, the present descriptive findings provide useful clinical insights. For patients with developmental epilepsy, a comprehensive preoperative evaluation focusing on lesion extent and surgical resectability is essential. The intraoperative use of neuronavigation and electrophysiological monitoring should be standardized to maximize the completeness of disconnection. In addition, intensive postoperative follow-up is warranted to facilitate the early detection and timely management of complications such as hydrocephalus, thereby reducing postoperative recurrence and optimizing long-term outcomes.

Owing to the limited sample size of this study, multivariate analysis was not performed to identify independent prognostic factors. Further studies with larger cohorts are needed to validate these findings.

Regarding postoperative functional outcomes, McNemar test results showed no statistically significant differences in independent ambulation, voluntary hand movement, or speech function between preoperative and postoperative assessments (all *P* > 0.05). These findings should be interpreted with caution in the context of patient selection and clinical expectations. Most patients in this cohort had preexisting functional deficits prior to surgery, as they were selected for hemispheric disconnection surgery based on the presence of unilateral hemispheric lesions associated with established neurological impairment. In such cases, surgery is not intended to restore lost function but rather to prevent further deterioration and preserve the existing function of the contralateral hemisphere. The absence of significant postoperative functional decline is clinically meaningful, as it suggests that hemispheric disconnection surgery successfully preserves the functional capacity of the unaffected hemisphere despite extensive surgical intervention. Previous studies have shown that functional outcomes after hemispheric disconnection surgery are mainly determined by the preoperative functional status of the contralateral hemisphere and the degree of functional compensation achieved through neuroplasticity [20, 21]. Patients with established contralateral deficits prior to surgery typically maintain stable functional levels postoperatively, whereas those with preserved contralateral function may experience varying degrees of functional adaptation and compensation over time [22]. The absence of significant functional change in our cohort likely reflects baseline functional status and supports the notion that hemispheric disconnection surgery achieves its primary therapeutic goal of seizure control while maintaining functional stability in appropriately selected patients.

Abundant clinical evidence indicates that the location and burden of epileptiform discharges are of great value in the preoperative evaluation of epilepsy and can provide critical information for localization and quantitative analysis [11, 23]. EEG is also important in the preoperative assessment for hemispheric disconnection surgery; however, its clinical interpretation has specific characteristics, as the presence of contralateral or generalized epileptiform discharges preoperatively is not a contraindication to surgery [13, 24]. In this small-sample cohort, although 11 patients (68.8%) had preoperative contralateral epileptiform discharges, including 2 patients with postoperative seizure recurrence (co-occurrence rate: 12.5%), statistical analysis showed no significant association between preoperative contralateral discharges and prognosis. Further analysis suggested that recurrence was mainly related to etiological and surgery-related factors rather than the presence of contralateral epileptiform discharges per se.

For postoperative EEG in epilepsy, a reduction in epileptiform discharges is generally associated with a favorable prognosis, whereas epileptiform discharges are positively correlated with the risk of seizure recurrence [25]. Discontinuation of ASMs may be considered only when the EEG shows no epileptiform discharges for at least 6 months to 1 year in seizure-free postoperative patients [26]. However, a marked increase in abundant discharges over the ipsilateral hemisphere is commonly observed after hemispheric disconnection surgery, and decisions regarding ASM tapering or discontinuation should not be based solely on the presence of epileptiform discharges [27].

In this study, only 4 patients showed a reduction in epileptiform discharges in the ipsilateral hemisphere at 1 month postoperatively, which may be attributed to transient cerebral ischemia and hypoxia, as well as reduced neuronal activity secondary to surgical injury and inflammatory responses [28]. Most patients exhibited increased epileptiform discharges with higher amplitude and reduced frequency in the ipsilateral hemisphere within 1 year after surgery, accompanied by the emergence of burst-suppression-like patterns or electrographic seizures [29, 30]. Although no significant association was found between the burst-suppression-like pattern and surgical prognosis in the present cohort (*P* > 0.05), the co-occurrence rate of the burst-suppression-like pattern and postoperative seizure freedom was 68.8% (11/16). Moreover, 11 of the 14 patients undergoing ASM tapering exhibited a burst-suppression-like pattern. Previous studies by Wennberg [31] and Joshi [32] have demonstrated that the isolated cortex after functional disconnection exhibits a characteristic burst-suppression-like pattern, suggesting that this EEG pattern may reflect the physiological state of the disconnected cortex. However, we acknowledge that without systematic quantitative postoperative MRI assessment of disconnection completeness, a definitive causal relationship between the burst-suppression-like pattern and anatomical disconnection status cannot be established. The clinical observation that patients with a burst-suppression-like pattern achieved favorable seizure outcomes supports the hypothesis that this pattern reflects functional isolation of the epileptogenic cortex, but this requires validation through comprehensive imaging correlation in future studies. During long-term follow-up, the number and amplitude of epileptiform discharges in the ipsilateral hemisphere gradually decreased, and the discharges became more stable. The burst-suppression-like pattern disappeared, or its proportion decreased in the majority of cases (9/12, 75.0%), suggesting that the isolated cerebral cortex gradually achieves a new equilibrium state [33]. Thus, epileptiform discharges in the ipsilateral hemisphere exhibit dynamic changes following hemispheric disconnection surgery.

The occurrence of a burst-suppression-like pattern in the ipsilateral hemisphere after hemispheric disconnection surgery may involve several mechanisms. The core mechanism is that the disconnected cerebral cortex loses rhythmic drive and modulatory input from the thalamus, is unable to maintain stable and desynchronized activity, and consequently enters an abnormal, highly synchronized and imbalanced state [34, 35]. Meanwhile, reorganization of the local neural network in the ipsilateral hemisphere may lead to a marked disturbance in the excitation–inhibition balance. Reduced inhibition from GABAergic interneurons and increased excitation from glutamatergic neurons jointly lower the burst threshold [36]. In addition, cerebral blood perfusion and glucose metabolism in the ipsilateral hemisphere are significantly reduced (blood flow decreased by 30–50%), and insufficient adenosine triphosphate (ATP) production fails to sustain continuous neuronal electrical activity. Together with abnormal neurotransmitter levels and disrupted ionic homeostasis, these changes further contribute to the formation and maintenance of the burst-suppression-like pattern [34].

EEG background activity serves as a core indicator for evaluating cerebral functional status [37]. Postoperatively, the EEG background in the ipsilateral hemisphere in the present cohort was dominated by slow waves and low-voltage activity. As illustrated in Fig. 1, the EEG on this side exhibited a dynamic pattern characterized by initial slow waves, subsequent low-voltage activity, and later re-emergence of slow waves. This trend not only reflects the functional status of the ipsilateral hemisphere after surgery but also indirectly indicates the completeness of hemispheric disconnection [33]. Early postoperatively, the background activity of the ipsilateral hemisphere was dominated by slow waves, which are closely related to acute disruption of thalamocortical projection fibers and early functional suppression caused by transient metabolic disturbances [38, 39]. Approximately 1 year after surgery, the background activity of the ipsilateral hemisphere was mainly characterized by low-voltage activity. The underlying mechanism is thought to involve loss of afferent drive to cortical electrical activity in a permanently deafferented state, together with chronic neurodegenerative changes and metabolic reorganization [38, 39]. Long-term follow-up showed that the background activity of the ipsilateral hemisphere remained characterized by low-voltage activity. At this stage, local intracortical circuits may regain weak autonomous activity; however, owing to the permanent loss of rhythmic drive from the thalamus and brainstem, the activity eventually stabilizes into low-voltage patterns. This phenomenon has been directly demonstrated in a human clinical study by Colombo [33]. In the present cohort, although no statistical association was found between low-voltage activity and the burst-suppression-like pattern, they showed a high co-occurrence rate, and both indicated profound suppression of cortical function [40]. Therefore, the intrinsic relationship between them requires further analysis and validation in larger cohort studies. In contrast, the EEG background activity of the contralateral hemisphere gradually returned to normal physiological rhythms in most patients, and partial recovery of motor and language functions was observed postoperatively. These findings suggest that surgery alleviated the abnormal inhibitory influence of the affected hemisphere on the unaffected hemisphere, thereby preserving and potentially improving contralateral hemispheric function [41].

The association between contralateral hemispheric epileptiform discharges and prognosis after hemispheric disconnection surgery remains controversial [27, 42]. When evaluating their prognostic significance, the dynamic changes in discharges before and after surgery should be considered. Persistent or progressively worsening postoperative contralateral epileptiform discharges may indicate a poorer prognosis. Possible mechanisms underlying the increase in contralateral epileptiform discharges postoperatively include: (1) following the removal of abnormal inhibitory influence from the ipsilateral hemisphere, previously suppressed excitability in the contralateral hemisphere may be unmasked, or preexisting epileptiform discharges previously obscured by ipsilateral activity may become apparent [43, 44]; (2) instability during neural network reorganization in the contralateral hemisphere may give rise to abnormal, highly synchronized circuits, resulting in transient epileptiform discharges [45]; and (3) surgical stress may lower the global seizure threshold [46]. In the present cohort, most preoperatively present contralateral discharges gradually disappeared postoperatively, suggesting that this phenomenon may be attributed to the removal of the influence exerted by the ipsilateral epileptogenic lesion on the contralateral hemisphere. Only three patients showed persistent epileptiform discharges during postoperative follow-up. Among them, one patient had preexisting discharges, in whom potential epileptogenicity should be considered. The remaining two cases were associated with complications or incomplete disconnection, and their epileptiform discharges showed a decreasing trend after reoperation. Continued follow-up is warranted to monitor the dynamic evolution of epileptiform discharges.

This study is the first to systematically observe and summarize the dynamic evolution of EEG following hemispheric disconnection surgery and to preliminarily explore its potential underlying mechanisms. The results demonstrated that both the ipsilateral and contralateral hemispheres exhibited dynamic EEG changes postoperatively in patients with DRE. A burst-suppression-like pattern in the ipsilateral hemisphere was relatively common, whereas no EEG features were found to be significantly associated with epilepsy prognosis. These findings may provide useful implications for clinicians in postoperative prognostic assessment using long-term VEEG.

However, this study has several limitations that should be acknowledged. First, this was a single-center study with a small sample size, which precluded multivariate regression analysis and resulted in limited statistical power. Although several intergroup differences did not reach statistical significance, the possibility of false-negative findings due to insufficient sample size cannot be excluded, thereby limiting the external validity of the present conclusions. The post-hoc power analysis (power = 17.2%, Type II error > 80.0%) quantified the extent of this limitation. Such extremely low statistical power prevents definitive conclusions regarding the prognostic value of EEG characteristics. Future studies with larger sample sizes (*n* ≥ 100–150) are needed to achieve adequate statistical power (≥ 80.0%) for detecting clinically meaningful associations (OR ≥ 3.0) and for establishing reliable prognostic factors following hemispheric disconnection surgery.

Second, the duration of postoperative EEG monitoring was limited to ≥ 3 h, which is substantially shorter than the ≥ 24-hour standard used for preoperative evaluation. This represents a key methodological limitation affecting the reliability of interictal epileptiform discharge quantification in several ways: (1) the shorter recording duration may underestimate the true burden of interictal epileptiform discharges; (2) the detection rate of electrographic seizures may be reduced, as shorter recordings lower the likelihood of capturing subclinical seizure events; and (3) the quantification of burst-suppression-like pattern burden (suppression epoch ratio) may be subject to sampling bias. However, several factors partially mitigate these limitations: (1) the primary objective of this study was to characterize qualitative EEG patterns (background activity, interictal, and ictal patterns) rather than to perform precise quantitative burden assessment; qualitative pattern identification is less dependent on recording duration than frequency-based measurements; (2) all recordings included video monitoring, enabling correlation of EEG findings with clinical manifestations and reducing the risk of missing clinically significant events; and (3) discharge quantification was standardized as frequency per hour, allowing comparison across recordings of different durations. Nevertheless, we emphasize that the findings regarding discharge burden and electrographic seizure frequency should be interpreted as preliminary estimates rather than definitive measurements. Future studies with extended postoperative VEEG monitoring (≥ 24 h) are strongly recommended to validate and refine the quantitative findings presented in this study.

Third, the study population was predominantly composed of young patients, which limits the generalizability of our findings to older pediatric and adult populations. As a result, age-related differences in postoperative EEG evolution could not be adequately assessed, and the potential impact of age-related selection bias on the study results cannot be excluded.

Fourth, this study had limitations in neuroimaging integration: (1) the absence of diffusion tensor imaging (DTI) precluded quantification of residual neural connectivity and tractography-based assessment of disconnection completeness; (2) the lack of postoperative FDG-PET data limited the evaluation of metabolic changes in the ipsilateral hemisphere and their correlation with EEG patterns, particularly the metabolic basis of burst-suppression-like patterns; and (3) systematic quantitative postoperative MRI assessment of disconnection completeness was not performed uniformly across all patients. Although qualitative postoperative MRI was available, the absence of standardized volumetric analysis or graded disconnection scoring limits the ability to establish robust anatomical–EEG correlations. This represents a significant limitation when interpreting the burst-suppression-like pattern as a marker of disconnection completeness. Future studies incorporating comprehensive postoperative neuroimaging protocols, including DTI tractography, quantitative volumetric MRI, and postoperative FDG-PET, are essential to validate the relationship between anatomical disconnection status and postoperative EEG characteristics.

## Conclusions

Hemispheric disconnection surgery achieves excellent seizure control in patients with DRE secondary to hemispheric lesions. Following surgery, the ipsilateral and contralateral hemispheres exhibit distinct and dynamic EEG evolutionary patterns. The ipsilateral hemisphere is characterized by a reversible burst-suppression-like pattern and a trajectory of background activity transitioning from slow waves to low-voltage activity and eventually returning to slow waves, reflecting progressive cortical functional isolation and subsequent network reorganization. In contrast, epileptiform discharges in the contralateral hemisphere gradually diminish or resolve in most patients, accompanied by normalization of background activity. In this small exploratory cohort, no significant association was identified between postoperative EEG characteristics and seizure prognosis; however, extremely low statistical power (< 20%) precludes definitive conclusions regarding the prognostic value of EEG features. These findings provide a preliminary framework for interpreting postoperative EEG following hemispheric disconnection surgery and may guide ASM management decisions. The burst-suppression-like pattern should be recognized as a physiological marker of cortical isolation rather than an indicator of poor outcome. Future large-sample, multicenter, prospective studies with standardized quantitative neuroimaging correlation and extended EEG monitoring are essential to validate these observations and establish reliable EEG-based prognostic criteria.

## Supplementary Information

Below is the link to the electronic supplementary material.

Supplementary Material 1

## Data Availability

The data that support the findings of this study are available from the corresponding author upon reasonable request.

## Abbreviations

ASMs: Anti-seizure medications
ATP: Adenosine triphosphate
DRE: Drug-resistant epilepsy
DTI: Diffusion tensor imaging
EEG: Electroencephalogram
ECG: Electrocardiogram
EMG: Electromyogram
FLAIR: Fluid-attenuated inversion recovery
FDG-PET: Fluorodeoxyglucose-positron emission tomography
HF: High-frequency filter
HARNESS: Harmonized Neuroimaging of Epilepsy Structural Sequences
ILAE: International League Against Epilepsy
LF: Low-frequency filter
LVPS: Lateral ventriculo-peritoneal shunt
MRI: Magnetic resonance imaging
T1WI: T1-weighted imaging
T2WI: T2-weighted imaging
VEEG: Video-electroencephalogram

## References

[1] Dorfer C, Czech T, Dressler A, Gröppel G, Mühlebner-Fahrngruber A, Novak K, et al. Vertical perithalamic hemispherotomy: a single-center experience in 40 pediatric patients with epilepsy. Epilepsia. 2013;54(11):1905–12. 10.1111/epi.12394. 24116936 10.1111/epi.12394

[2] Jehi L. Advances in Therapy for Refractory Epilepsy. Annu Rev Med. 2025;76(1):389–402. 10.1146/annurev-med-050522-034458. 39532109 10.1146/annurev-med-050522-034458

[3] Dai L, Huang J, Shi XJ, Sun XQ, Shen KF, Zhu M. Efficacy analysis of hemispheric disconnection for drug-resistant epilepsy. J Epilepsy Electroneurophysiol. 2023;32(2):81–86. (in Chinese). 10.19984/j.cnki.1674-8972.2023.02.04.

[4] Qu XP, Qu Y, Wang C, Liu B. Long-term cognitive improvement after functional hemispherectomy. World Neurosurg. 2020;135:e520-e526. 10.1016/j.wneu.2019.12.058. 10.1016/j.wneu.2019.12.058 31863898

[5] Ramantani G, Cserpan D, Tisdall M, Otte WM, Dorfmüller G, Cross JH, et al. Determinants of Functional Outcome after Pediatric Hemispherotomy. Ann Neurol. 2024;95(2):377–87. 10.1002/ana.26830. 37962290 10.1002/ana.26830

[6] Liu JT, Liu B, Zhang H. Surgical versus medical treatment of drug-resistant epilepsy: A systematic review and meta-analysis. Epilepsy Behav. 2018;82:179–88. 10.1016/j.yebeh.2017.11.012. 29576434 10.1016/j.yebeh.2017.11.012

[7] Rugg-Gunn F, Miserocchi A, McEvoy A. Epilepsy surgery. Pract Neurol. 2020;20(1):4–14. 10.1136/practneurol-2019-002192. 31420415 10.1136/practneurol-2019-002192

[8] Berg AT, Berkovic SF, Brodie MJ, Buchhalter J, Cross JH, van Emde Boas W, et al. Revised terminology and concepts for organization of seizures and epilepsies: report of the ILAE Commission on Classification and Terminology, 2005–2009. Epilepsia. 2010;51(4):676–85. 10.1111/j.1528-1167.2010.02522.x. 20196795 10.1111/j.1528-1167.2010.02522.x

[9] Faul F, Erdfelder E, Lang AG, Buchner A G*Power 3: a flexible statistical power analysis program for the social, behavioral,and biomedical sciences. Behav Res Methods. 2007;39(2):175–91. 10.3758/bf03193146. 17695343 10.3758/bf03193146

[10] Landis JR, Koch GG. The measurement of observer agreement for categorical data. Biometrics. 1977;33(1):159–74. 843571

[11] Beniczky S, Trinka E, Wirrell E, Abdulla F, Al Baradie R, Alonso Vanegas M, et al. Updated classification of epileptic seizures: Position paper of the International League Against Epilepsy. Epilepsia. 2025;66(6):1804–23. 10.1111/epi.18338. 40264351 10.1111/epi.18338 PMC12169392

[12] Rasmussen T. Hemispherectomy for seizures revisited. Can J Neurol Sci. 1983;10(2):71–8. 10.1017/s0317167100044668. 6861011 10.1017/s0317167100044668

[13] Ko PY, Barry D, Shurtleff H, Hauptman JS, Marashly A. Prognostic Value of Preoperative and Postoperative Electroencephalography Findings in Pediatric Patients Undergoing Hemispheric Epilepsy Surgery. World Neurosurg. 2022;167:e1154–62. 10.1016/j.wneu.2022.08.138. 36084916 10.1016/j.wneu.2022.08.138

[14] Weil AG, Lewis EC, Ibrahim GM, Kola O, Tseng CH, Zhou X, et al. Hemispherectomy Outcome Prediction Scale: Development and validation of a seizure freedom prediction tool. Epilepsia. 2021;62(5):1064–73. 10.1111/epi.16861. 33713438 10.1111/epi.16861

[15] Ramantani G, Bulteau C, Cserpan D, Otte WM, Dorfmüller G, Cross JH, et al. Not surgical technique, but etiology, contralateral MRI, prior surgery, and side of surgery determine seizure outcome after pediatric hemispherotomy. Epilepsia. 2023;64(5):1214–24. 10.1111/epi.17574. 36869851 10.1111/epi.17574

[16] Boshuisen K, van Schooneveld MM, Leijten FS, de Kort GA, van Rijen PC, Gosselaar PH, et al. Contralateral MRI abnormalities affect seizure and cognitive outcome after hemispherectomy. Neurology. 2010;75(18):1623–30. 10.1212/WNL.0b013e3181fb4400. 21041785 10.1212/WNL.0b013e3181fb4400

[17] Althausen A, Gleissner U, Hoppe C, Sassen R, Buddewig S, von Lehe M, et al. Long-term outcome of hemispheric surgery at different ages in 61 epilepsy patients. J Neurol Neurosurg Psychiatry. 2013;84(5):529–36. 10.1136/jnnp-2012-303811. 23268362 10.1136/jnnp-2012-303811

[18] Volpon Santos M, Teixeira TL, Ioriatti ES, Thome U, Paula de Andrade Hamad A, Machado HR. Risk factors and results of hemispherotomy reoperations in children. Neurosurg Focus. 2020;48(4):E5. 10.3171/2020.1.FOCUS19944. 32234979 10.3171/2020.1.FOCUS19944

[19] Pepi C, De Benedictis A, Rossi-Espagnet MC, Cappelletti S, Da Rold M, Falcicchio G, et al. Hemispherotomy in Infants with Hemimegalencephaly: Long-Term Seizure and Developmental Outcome in Early Treated Patients. Brain Sci. 2022;13(1):73. 10.3390/brainsci13010073. 36672056 10.3390/brainsci13010073 PMC9856354

[20] Weil AG, Fallah A, Wang S, Ibrahim GM, Elkaim LM, Jayakar P, et al. Functional hemispherectomy: can preoperative imaging predict outcome? J Neurosurg Pediatr. 2020;25(6):567–73. 10.3171/2019.12.PEDS19370. 33988937 10.3171/2019.12.PEDS19370

[21] Höller Y, Versace V, Trinka E, Nardone R. Functional connectivity after hemispherectomy. Quant Imaging Med Surg. 2020;10(5):1174–8. 10.21037/qims.2020.03.17. 32489942 10.21037/qims.2020.03.17 PMC7242305

[22] Bergeron D, Barthélemy D, Hadjinicolaou A, Bonizzato M, Martinez M, Dancause N, et al. Motor Recovery After a Hemispherectomy: Review of Mechanisms and the Potential of Neuromodulation to Enhance Motor Outcomes. J Child Neurol. 2026;41(6):892–910. 10.1177/08830738251413830. 41686708 10.1177/08830738251413830 PMC13219769

[23] Karoly PJ, Freestone DR, Boston R, Grayden DB, Himes D, Leyde K, et al. Interictal spikes and epileptic seizures: their relationship and underlying rhythmicity. Brain. 2016;139(Pt 4):1066–78. 10.1093/brain/aww019. 26912639 10.1093/brain/aww019

[24] Abraham AP, Thomas MM, Mathew V, Muthusamy K, Yoganathan S, Jonathan GE, et al. EEG lateralization and seizure outcome following peri-insular hemispherotomy for pediatric hemispheric epilepsy. Childs Nerv Syst. 2019;35(7):1189–95. 10.1007/s00381-019-04067-6. 30701299 10.1007/s00381-019-04067-6

[25] Rathore C, Radhakrishnan K. Prognostic significance of interictal epileptiform discharges after epilepsy surgery. J Clin Neurophysiol. 2010;27(4):255–62. 10.1097/WNP.0b013e3181eaa5fa. 20634715 10.1097/WNP.0b013e3181eaa5fa

[26] Hildebrandt M, Schulz R, Hoppe M, May T, Ebner A. Postoperative routine EEG correlates with long-term seizure outcome after epilepsy surgery. Seizure. 2005;14(7):446–51. 10.1016/j.seizure.2005.07.007. 16139529 10.1016/j.seizure.2005.07.007

[27] Alzahrany M, Alnakhli R, Bingaman W, Wyllie E, Moosa AN. Epileptiform abnormalities in the disconnected hemisphere are common in seizure-free patients after hemispherectomy. Epileptiform abnormalities in the disconnected hemisphere are common in seizure-free patients after hemispherectomy. Epileptic Disord. 2022;24(5):857–66. 10.1684/epd.2022.1464. 35872623 10.1684/epd.2022.1464

[28] Wu YX, Bai Y, Jiang Z, Fu XX, Liang F, Han RQ, et al. Advances in diagnosis and treatment of cerebral vasospasm following subarachnoid hemorrhage. Int J Anesthesiology Resusc. 2022;43(10):1097–102. (in Chinese). 10.3760/cma.j.cn321761-20210826-00661.

[29] Alzahrany M, Alnakhli R, Bingaman W, Wyllie E, Moosa AN. Epileptiform abnormalities in the disconnected hemisphere are common in seizure-free patients after hemispherectomy. Epileptiform abnormalities in the disconnected hemisphere are common in seizure-free patients after hemispherectomy. Epileptic Disord. 2022;24(5):857–66. 10.1684/epd.2022.1464. 35872623 10.1684/epd.2022.1464

[30] Alzahrany M, Alnakhli R, Bingaman W, Wyllie E, Moosa AN. Epileptiform abnormalities in the disconnected hemisphere are common in seizure-free patients after hemispherectomy. Epileptic Disord. 2022;24(5):857–66. 10.1684/epd.2022.1464. 35872623 10.1684/epd.2022.1464

[31] Wennberg RA, Quesney LF, Villemure JG. Epileptiform and non-epileptiform paroxysmal activity from isolated cortex after functional hemispherectomy. Electroencephalogr Clin Neurophysiol. 1997;102(5):437–42. 10.1016/s0921-884x(97)96047-1. 9191587 10.1016/s0921-884x(97)96047-1

[32] Joshi S, Chandran AS, Ochi A, Otsubo H, Ibrahim GM, Rutka JT, et al. Burst suppression pattern on Stereo EEG of the isolated cortex after functional disconnection. Clin Neurophysiol. 2025;173:216–8. 10.1016/j.clinph.2025.03.028. 40184892 10.1016/j.clinph.2025.03.028

[33] Colombo MA, Favaro J, Mikulan E, Pigorini A, Zauli FM, Sartori I, et al. Hemispherotomy leads to persistent sleep-like slow waves in the isolated cortex of awake humans. PLoS Biol. 2025;23(10):e3003060. 10.1371/journal.pbio.3003060. 41100501 10.1371/journal.pbio.3003060 PMC12530565

[34] Ching S, Purdon PL, Vijayan S, Kopell NJ, Brown EN. A neurophysiological-metabolic model for burst suppression. Proc Natl Acad Sci U S A. 2012;109(8):3095–100. 10.1073/pnas.1121461109. 22323592 10.1073/pnas.1121461109 PMC3286963

[35] Niedermeyer E. The burst-suppression electroencephalogram. Am J Electroneurodiagnostic Technol. 2009;49(4):333–41. 20073416

[36] Liou JY, Maciver MB, Sleigh JW. Solving the enigma of burst suppression. Br J Anaesth. 2025;134(4):900–2. 10.1016/j.bja.2024.12.013. 40118581 10.1016/j.bja.2024.12.013 PMC11947557

[37] Liu XY. Clinical Electroencephalography. 2nd ed. Beijing: People’s Medical Publishing House; 2017. (in Chinese).

[38] Steriade M, McCormick DA, Sejnowski TJ. Thalamocortical oscillations in the sleeping and aroused brain. Science. 1993;262(5134):679–85. 10.1126/science.8235588. 8235588 10.1126/science.8235588

[39] Schramm J, Behrens E, Entzian W. Hemispherical deafferentation: an alternative to functional hemispherectomy. Neurosurgery. 1995;36(3):509–16. 10.1227/00006123-199503000-00010. 7753351 10.1227/00006123-199503000-00010

[40] Brenner RP. The interpretation of the EEG in stupor and coma. Neurologist. 2005;11(5):271–84. 10.1097/01.nrl.0000178756.44055.f6. 16148734 10.1097/01.nrl.0000178756.44055.f6

[41] KRYNAUW RA. Infantile hemiplegia treated by removing one cerebral hemisphere. J Neurol Neurosurg Psychiatry. 1950;13(4):243–67. 10.1136/jnnp.13.4.243. 14795238 10.1136/jnnp.13.4.243 PMC498647

[42] Shi ZM, Zhang K, Zhang JG, Ma YS, Sang L, Zheng Z, et al. Hemispherectomy for intractable epilepsy: a follow-up study. Chin J Neurosurg. 2016;32(10):984–8. (in Chinese). 10.3760/cma.j.issn.1001-2346.2016.10.004.

[43] Rasmussen T. Hemispherectomy for seizures revisited. Can J Neurol Sci. 1983;10(2):71–8. 10.1017/s0317167100044668. 6861011 10.1017/s0317167100044668

[44] Borne A, Bulteau C, Ferrand-Sorbets S, Dorfmüller G, Baciu M, Perrone-Bertolotti M. Evaluation of long-term cognitive organization after hemispherotomy in Rasmussen’s encephalitis: A behavioral and network-level perspective. Cortex. 2025;191:140–53. 10.1016/j.cortex.2025.07.014. 40840189 10.1016/j.cortex.2025.07.014

[45] Petrik S, San Antonio-Arce V, Steinhoff BJ, Syrbe S, Bast T, Scheiwe C, et al. Epilepsy surgery: Late seizure recurrence after initial complete seizure freedom. Epilepsia. 2021;62(5):1092–104. 10.1111/epi.16893. 33778964 10.1111/epi.16893

[46] Vezzani A, French J, Bartfai T, Baram TZ. The role of inflammation in epilepsy. Nat Rev Neurol. 2011;7(1):31–40. 10.1038/nrneurol.2010.178. 21135885 10.1038/nrneurol.2010.178 PMC3378051

