# Supplementary material for: Characteristics of electroencephalographic changes after hemispheric disconnection surgery in patients with drug-resistant epilepsy
Source: Acta Epileptol. 2026 Aug 1;8:28. doi: 10.1186/s42494-026-00269-z (PMC13428446; doi:10.1186/s42494-026-00269-z)
Supplement: Supplementary file 1 — Supplementary Material 1 [file 42494_2026_269_MOESM1_ESM.docx]

### ****Supplementary Table 1:**The demographic data and detailed clinical information of the patients**

| **Case No.** | **Gender** | **Handedness** | **Age at surgery** | **Duration of epilepsy (months)** | **Seizure type** | **MRI lesion side** | **PET-CT** | **Seizure etiology** | **The operative side** | **Follow-up duration**  **(months)** | **Outcome** | **Preoperative functional assessment** | | | **Postoperative functional recovery** | | |
| --- | --- | --- | --- | --- | --- | --- | --- | --- | --- | --- | --- | --- | --- | --- | --- | --- | --- |
|  |  |  |  |  |  |  |  |  |  |  |  | **Independent ambulation** | **Voluntary hand movement** | **Speech** | **Independent ambulation** | **Voluntary hand movement** | **Speech** |
| 1 | Female | Right | 2 years | 12 | Focal | Right | Hemispheric hypometabolism | Schizencephaly, Gray matter heterotop | Right | 34 | Engel I, medication reduction | Yes | Yes | Yes | Yes | No | No |
| 2 | Male | Ambiguous | 4 years 8 months | 6 | Focal, Generalized | Right | Hemispheric hypometabolism | Encephalomalacia with atrophy | Right | 46 | Engel I,medication reduction | Yes | Yes | Yes | Yes | Yes | Yes |
| 3 | Female | Ambiguous | 2 years 4 months | 24 | Focal | Left | Hemispheric hypometabolism | FCDI | Left | 38 | Engel I,medication reduction | No | No | No | Yes | No | No |
| 4 | Female | Ambiguous | 4 years 1 month | 46 | Focal | Right | Hemispheric hypometabolism | Encephalitis | Right | 32 | Engel I,medication reduction | Yes | Yes | No | Yes | Yes | Yes |
| 5 | Female | Ambiguous | 2 years 1 month | 14 | Generalized | Left | Hemispheric hypometabolism | FCDIB | Left | 33 | Engel I,medication reduction | No | No | No | No | No | No |
| 6 | Female | Right | 5 years 5 months | 12 | Focal | Right | Hemispheric hypometabolism | Hemorrhagic cerebral Infarction | Right | 34 | Engel I,medication reduction | No | No | No | No | No | No |
| 7 | Female | Left | 6 years 3 months | 60 | Generalized | Left | Hemispheric hypometabolism | FCDIB | Left | 52 | Engel I,medication reduction | Yes | Yes | No | Yes | Yes | Yes |
| 8 | Female | Right | 5 years 4 months | 10 | Focal | Right | Hemispheric hypometabolism | Polymicrogyria, FCD I | Right | 40 | Engel I,medication reduction | Yes | Yes | Yes | Yes | No | Yes |
| 9 | Male | Left | 6 years 2 months | 11 | Focal | Left | Hemispheric hypometabolism | FCD IIID+IB, Rasmussen's encephalitis | Left | 41 | Engel I,medication reduction | No | No | No | No | No | No |
| 10 | Female | Left | 2 years 4 months | 6 | Focal | Left | Hemispheric hypometabolism | Ulegyria, FCD IIID | Left | 43 | Engel I,medication reduction | Yes | Yes | No | Yes | No | No |
| 11 | Male | Right | 4 years 5 months | 48 | Focal | Right | Hemispheric hypometabolism | Pachygyria | Right | 45 | Engel I,medication reduction | No | No | No | No | No | No |
| 12 | Female | Left | 1 year 8 months | 17 | Focal | Left | Hemispheric hypometabolism | Pachygyria | Left | 45 | Engel I,medication reduction | No | No | No | Yes | Yes | No |
| 13 | Male | Right | 6 years 5 months | 57 | Focal | Right | Hemispheric hypometabolism | Encephalomalacia, Status post cerebral hemorrhage | Right | 55 | Engel I,medication reduction | Yes | Yes | Yes | Yes | Yes | Yes |
| 14 | Male | Ambiguous | 4 months | 2 | Focal | Left | Hemispheric Hypometabolism | Pachygyria, FCD IIa | Left | 48 | Transient recurrence; Engel I at last follow-up | No | No | No | No | No | No |
| 15 | Female | Right | 3 years 10 months | 24 | Focal | Right | Hemispheric Hypometabolism | Sturge-Weber syndrome | Right | 45 | Transient recurrence; Engel I at last follow-up | Yes | Yes | Yes | Yes | No | No |
| 16 | Male | Right | 3 years 10 months | 5 | Focal | Right | Hemispheric Hypometabolism | Cortical malformation, FCD Ib | Right | 27 | Engel I,medication reduction | Yes | Yes | Yes | Yes | Yes | Yes |

****Supplementary Table 2**: Preoperative VEEG**

| **Case No.** | **Background** | | **Interictal activity** | | | **Ictal activity** |
| --- | --- | --- | --- | --- | --- | --- |
|  | **‌Ipsilateral** | **Contralateral** | **‌Ipsilateral** | **Contralateral** | **Generalized discharges** |  |
| 1 | Slow waves | Normal | Abundant regional slow waves and discharges |  |  | Ipsilateral focal-onset seizures |
| 2 | Slow waves | Slow waves | Abundant multifocal discharges | Abundant multifocal discharges | Yes | Ipsilateral focal-onset seizures+generalized seizures |
| 3 | Slow waves | Slow waves | Abundant multifocal slow waves and discharges |  |  | Ipsilateral focal-onset seizures |
| 4 | Slow waves | Normal | Abundant regional slow waves and discharges |  |  | Ipsilateral focal-onset seizures |
| 5 | Normal | Normal | Abundant multifocal slow waves and discharges |  | Yes | Generalized seizures |
| 6 | Low-voltage activities | Normal | Occasional regional discharges | Occasional regional discharges |  | Ipsilateral focal-onset seizures |
| 7 | Slow waves | Normal | Abundant regional slow waves and discharges | Occasional regional discharges |  | Generalized seizures |
| 8 | Slow waves | Normal | Abundant multifocal discharges | Occasional regional discharges | Yes | Ipsilateral focal-onset seizures |
| 9 | Slow waves | Normal | Abundant multifocal slow waves and discharges | Occasional regional slow waves and discharges |  | Ipsilateral focal-onset seizures |
| 10 | Slow waves | Normal | Abundant multifocal slow waves and discharges |  |  | Ipsilateral focal-onset seizures |
| 11 | Slow waves | Slow waves | Abundant regional discharges |  |  | Ipsilateral focal-onset seizures |
| 12 | Normal | Normal | Abundant regional discharges | Occasional regional discharges |  | Ipsilateral focal-onset seizures |
| 13 | Low-voltage activities | Normal | Abundant multifocal discharges | Abundant regional discharges |  | Ipsilateral focal-onset electrographic seizures |
| 14 | Slow waves | Normal | Abundant multifocal slow waves and discharges | Abundant regional discharges |  | Ipsilateral focal-onset seizures |
| 15 | Slow waves | Normal | Abundant multifocal slow waves and discharges | Occasional regional discharges |  | Ipsilateral focal-onset seizures |
| 16 | Slow waves | Normal | Abundant regional slow waves and discharges | Occasional regional discharges |  | Ipsilateral focal-onset seizures |

**Note**: Background slow waves refer to low- to moderate-amplitude theta and delta waves; background low-voltage activities refer to theta and delta waves with amplitudes <20 μV. Quantitative criteria for interictal epileptiform discharges: rare, <1/hour; occasional, ≥1/hour but <1/minute; frequent, ≥1/minute but <1/10 seconds; abundant, ≥1/10 seconds^[45]^.

****Supplementary Table 3**:** Postoperative VEEG

| Case No. | Background | | Interictal activity | | Postoperative Seizures |
| --- | --- | --- | --- | --- | --- |
|  | ‌Ipsilateral  (3m → 1y → 2y) | Contralateral  (3m → 1y → 2y) | ‌Ipsilateral  (3m → 1y → 2y) | Contralateral  (3m → 1y → 2y) |  |
| 1 | Low-voltage activities→ Low-voltage activities→ Slow waves |  | Occasional discharges→ Abundant discharges, burst-suppression-like pattern→ Abundant discharges, burst-suppression-like pattern resolved |  | Ipsilateral electrographic seizures at 1 year post-operation |
| 2 | Low-voltage activities→ Slow waves→ Slow waves | Slow waves reduction → normal → normal | Rare discharges→ Frequent discharges→ Frequent discharges | Occasional discharges → Discharges resolved | None |
| 3 | Slow waves→ Slow waves → Slow waves | Slow waves reduction → normal → normal | Abundant discharges, burst-suppression-like pattern→ Abundant discharges, more pronounced burst-suppression-like pattern→ Abundant discharges, attenuated burst-suppression-like pattern |  | None |
| 4 | Slow waves→ Low-voltage activities→ Slow waves |  | Abundant discharges, burst-suppression-like pattern → Abundant discharges, more pronounced burst-suppression-like pattern → Abundant discharges, attenuated burst-suppression-like pattern | Occasional discharges → Discharges resolved | None |
| 5 | Low-voltage activities→ Low-voltage activities→  Low-voltage activities |  | Abundant discharges, burst-suppression-like pattern→ Abundant discharges, burst-suppression-like pattern→ Abundant discharges, burst-suppression-like pattern | Rare discharges → Discharges resolved | None |
| 6 | Low-voltage activities→ Low-voltage activities→  Low-voltage activities |  | Occasional discharges → Rare discharges → Rare discharges |  | None |
| 7 | Low-voltage activities→ Slow waves → Slow waves |  | Discharges resolved → Frequent discharges → Frequent discharges | Occasional discharges →Frequent discharges → Frequent discharges | None |
| 8 | Slow waves→ Low-voltage activities→ Slow waves |  | Frequent discharges, burst-suppression-like pattern → Abundant discharges, bburst-suppression-like pattern→ Abundant discharges, burst-suppression-like pattern resolved | Occasional discharges → Discharges resolved | None |
| 9 | Slow waves→ Low-voltage activities→ Slow waves |  | Abundant discharges, burst-suppression-like pattern→ Abundant discharges, burst-suppression-like pattern→ Abundant discharges, attenuated burst-suppression-like pattern | Occasional discharges → Discharges resolved | None |
| 10 | Slow waves → Low-voltage activities→ Slow waves |  | Abundant discharges, burst-suppression-like pattern→ Abundant discharges, burst-suppression-like pattern→ Abundant discharges, attenuated burst-suppression-like pattern |  | None |
| 11 | Low-voltage activities→ Low-voltage activities→ Slow waves | Slow waves reduction → normal → normal | Frequent discharges, burst-suppression-like pattern→ Abundant discharges, more pronounced burst-suppression-like pattern→ Frequent discharges, burst-suppression-like pattern resolved |  | None |
| 12 | Slow waves→ Slow waves→ Slow waves |  | Abundant discharges, burst-suppression-like pattern→ Abundant discharges, burst-suppression-like pattern→ Frequent discharges, attenuated burst-suppression-like pattern | Discharges resolved | None |
| 13 | Low-voltage activities→ Low-voltage activities→  Low-voltage activities |  | Occasional discharges → Abundant discharges, burst-suppression-like pattern→Abundant discharges, burst-suppression-like pattern | Discharges resolved | None |
| 14 | Slow waves→ Low-voltage activities→  Low-voltage activities |  | Frequent discharges → Abundant discharges, burst-suppression-like pattern→Abundant discharges, burst-suppression-like pattern | Rare discharges → Occasional discharges → Rare discharges | Ipsilateral focal-onset seizures and electrographic seizure at 3 months post-operation; controlled after hydrocephalus shunt |
| 15 | Low-voltage activities→ Low-voltage activities→  Low-voltage activities |  | Frequent discharges → Abundant discharges → (Post 2nd op) Abundant discharges | Occasional discharges →Frequent discharges→ Occasional discharges | Ipsilateral focal-onset seizures at 1 year post-operation, with cessation after repeat disconnection surgery |
| 16 | Slow waves→ Slow waves → Slow waves |  | Abundant discharges, burst-suppression-like pattern→ Abundant discharges, burst-suppression-like pattern→ Frequent discharges, attenuated burst-suppression-like pattern | Discharges resolved | None |

**Note**: Background slow waves refer to low- to moderate-amplitude theta and delta waves; background low-voltage activities refer to theta and delta waves with amplitudes <20 μV.Quantitative criteria for interictal epileptiform discharges: rare, <1/hour; occasional, ≥1/hour but <1/minute; frequent, ≥1/minute but <1/10 seconds; abundant, ≥1/10 seconds^[45]^. For burst-suppression-like pattern, quantification is based on the suppression epoch ratio (suppression duration as a percentage of total recording time): abundant, suppression >50%; attenuated, ≥30% reduction in burst-suppression-like pattern amplitude ratio or ≥50% decrease in overall burst-suppression-like pattern burden.
